# Maximum Entropy Technique and Regularization Functional for Determining the Pharmacokinetic Parameters in DCE-MRI

**DOI:** 10.1007/s10278-022-00646-3

**Published:** 2022-05-26

**Authors:** Zahra Amini Farsani, Volker J Schmid

**Affiliations:** 1 Bayesian Imaging and Spatial Statistics Group, Institute of Statistics, Ludwig-Maximilian-Universität München, Ludwigstraße 33, 80539 Munich, Germany; 2 Statistics Department, School of Science, Lorestan University, 68151-44316 Khorramabad, Iran

**Keywords:** Maximum entropy technique, Arterial input function, Regularization Functional, Dynamic contrast-enhanced MRI, Gamma distribution, Pharmacokinetic parameters

## Abstract

This paper aims to solve the arterial input function (AIF) determination in dynamic contrast-enhanced MRI (DCE-MRI), an important linear ill-posed inverse problem, using the maximum entropy technique (MET) and regularization functionals. In addition, estimating the pharmacokinetic parameters from a DCE-MR image investigations is an urgent need to obtain the precise information about the AIF–the concentration of the contrast agent on the left ventricular blood pool measured over time. For this reason, the main idea is to show how to find a unique solution of linear system of equations generally in the form of $y=Ax+b,$ named an ill-conditioned linear system of equations after discretization of the integral equations, which appear in different tomographic image restoration and reconstruction issues. Here, a new algorithm is described to estimate an appropriate probability distribution function for AIF according to the MET and regularization functionals for the contrast agent concentration when applying Bayesian estimation approach to estimate two different pharmacokinetic parameters. Moreover, by using the proposed approach when analyzing simulated and real datasets of the breast tumors according to pharmacokinetic factors, it indicates that using Bayesian inference—that infer the uncertainties of the computed solutions, and specific knowledge of the noise and errors—combined with the regularization functional of the maximum entropy problem, improved the convergence behavior and led to more consistent morphological and functional statistics and results. Finally, in comparison to the proposed exponential distribution based on MET and Newton’s method, or Weibull distribution via the MET and teaching–learning-based optimization (MET/TLBO) in the previous studies, the family of Gamma and Erlang distributions estimated by the new algorithm are more appropriate and robust AIFs.

## Introduction

As a fast and noninvasive approach, dynamic contrast-enhanced magnetic resonance imaging (DCE-MRI) is widely used to analyze to quantitatively analyze perfusion in soft tissues in various clinical applications. These include the detection, characterization, and monitoring of different diseases for therapeutic purposes [1–6]. Typically, pharmacokinetic models are used in the quantitative analysis of DCE-MR images. For the DCE-MRI scan, an extracellular contrast agent with a low molecular weight such as gadolinium diethylenetriaminepentaacetic acid, Gd-DTPA, is injected. The in vivo concentration of the contrast agent (CA) in tissue over time is measured using T1-weighted images. Several pharmacokinetic models have been developed for the characterization of the signal intensity change over time. These models allow to quantify local physiologic features of the tissue, known as pharmacokinetic parameters [7].

Among pharmacokinetic models, the two-compartment model is the most popular [7]. In this model, the change in the contrast agent concentration is attributed to its transfer between two compartments: the blood plasma and the extravascular extracellular space (EES) of the tissue. The pharmacokinetic model can be determined as solution to an ordinary-differential equation (ODE) describing the exchange between the compartments [8–15].

An important term in the pharmacokinetic model is the arterial input function (AIF), that is CA concentration in the left ventricular blood pool over time. Despite the fact that the AIF itself has no clinical relevance, its precise calculation is of particular interest for proper estimation of pharmacokinetic parameters [16, 17]. Given the strong dependence of the determined rate constants on the AIF [18–22], their quantification in an absolute and reliable manner requires a precise measurement.

However, in many cases the direct measurement of the AIF from DCE-MRI images is not possible, as no large vessel is in the field of view, for example in breast scans. As a replacement, it has been proposed to use a simplified method such as a population averaged AIF. These AIFs include bi-exponential functions with parameters obtained by [23, 24] or a mix of the two Gaussian with an exponential [8–15]. The literature is split over the efficiency of population averaged AIF, with some authors reporting its ability to adequately estimating pharmacokinetic parameters [25, 26], while others raise concerns [27, 28].

In recent years, a couple of models have been developed to estimate the AIF from DCE-MRI scans without larger vessels in the field of view. The aim is to estimate the AIF together with the corresponding pharmacokinetic parameters from the CA signal over time [29–32]. For partial and fully automated AIF estimation, several different techniques have been proposed. Fan et al. [33] attempted to extract the AIF with a cluster method, using a manually marked region of interest (ROI). Reishofer et al. [34] proposed AIF extraction using classification based on criteria involving inherent arterial input features including an early bolus arrival and fast passage, as well as a high-contrast agent concentration.

In this paper, we propose a novel method for estimating the probability density function (PDF) of the AIF directly from measured concentration-time curves in enhanced tissues. Statistically speaking, the determination of the AIF from DCE-MRI scans is seen as the determination of the PDF of sample data. From a conceptual perspective, the choice of the type of distribution is an open problem. When available information is limited, e.g., sample size is small and/or has lower–order moments, an approach based on the maximum entropy (ME) principle can be the solution. Using all available data the maximum entropy distribution is the estimation with the smallest bias. Nevertheless, applying ME has a number of theoretical and practical restrictions.

Many nonparametric and parametric techniques have been proposed to estimate the probability density function of a random variable from observations. The maximum entropy technique (MET) is a widely used method to estimate and determine the probability density, with known high accuracy and efficiency. In MET an optimization problem is solved to obtain the unknown density. Jaynes [35] proposed the ME principle as a statistical inference method and stated that by employing this principle, a probability density function is selected that corresponds to the available knowledge and provides no unwarranted information. In this regard, among probability density functions meeting some constraints, the one with smaller entropy has more information, hence less uncertainty [35–38]. Over the past decade, there has been an extensive application of entropy maximization or similar approaches, including the determination of macromolecular structures and interactions [39–50] and the inference of signaling [51–53] and regulatory networks [54, 55] as well as the coding organization in neural populations [56–65] according to the analysis of DNA sequences (e.g., the identification of specific binding sites) [65–71]. In addition, MET is an often used tool for image reconstruction. This includes applications in radio astronomical interferometry, dealing, on a daily basis, with images with large dynamic ranges and up to one million pixels [72–75].

In a previous work, we proposed using a combination of MET and Newton’s method for AIF estimation and maximum a posterior (MAP) for estimation of the pharmacokinetic parameters [76]. In another study, we proposed two enhanced algorithms to estimate the AIF as a combination of Bayesian inference and optimization techniques. The first algorithm combines MET, teaching–learning-based optimization (TLBO) to assess the performance of observer in the classification tasks with existing data, and Bayesian methods to estimate the pharmacokinetic parameters [77]. Similar to other algorithms inspired by nature, TLBO is also a population-based approach and employs a population of solutions to obtain a global result [31, 78]. The second algorithm is the combination of MET, a concave optimization method, and the general regularization approach. In the present work, we propose to regularize the ME problem and the ill-conditioned linear system of equations in the pharmacokinetic model. In general, regularization is an appropriate method to find stable solution to ill-posed inverse problems [79].

The first proposed algorithm uses the Weibull distribution as most robust model for the AIF. In the proposed improved algorithm, via regularization the family of Gamma distributions is the best solution of the ME problem . To estimate physiological parameters, extensive investigation was performed on empirical data, such that a better understanding of the performance of the proposed method could be obtained. The previously analyzed data were provided by Paul Strickland Scanner Center, Mount Vernon Hospital, Northwood, UK [7]. The data were acquired according to the recommendations of [80]. Informed consent was obtained from all patients.

The following sections are organized as follows. “Methodology” gives a brief description of the methodology. The pharmacokinetic model for the analysis of DCE-MRI data is described. Then the MET for the ill-posed inverse problem is developed, following by the regularization of the ME problem. The TLBO algorithm is presented, followed by the Bayesian approach. In addition, some characteristics and flowchart of the proposed algorithm are also provided. In “Numerical Experiment”, the developed method is used to analyze the in vivo DCE-MRI data. “Discussion and Conclusions” provides concluding remarks.

## Methodology

We propose a novel algorithm combining the MET with regularization functionals, enabling us to estimate the PDF of the AIF along with the pharmacokinetic parameters in a similar method in a Bayesian framework. Compared to previous methods, the proposed algorithm does not consist of several phases, decreasing the computation time of the estimation. The proposed algorithm is a robust combination of MET, regularization, and the Bayesian estimation approach.

### Basic Model

#### Pharmacokinetic Model

Here, the popular pharmacokinetic model [81] is considered. The assumption of this model is that the CA resides in two compartments of the tissue, the vascular space and the extracellular extravascular space (EES), with exchange of CA between these two compartments. The exchange of CA in the tissue of interest ($C_{T}(t)$) can be described via an ODE [8–16],

1 $$\begin{aligned} \frac{dC_{T}(t)}{dt} =K_{a} C_{p} (t)-K_{b} C_{T} (t), \end{aligned}$$

where $C_{p} (t)$ gives the concentration of CA in the vascular blood pool, that is the AIF, $K_{a}$ and $K_{b}$ are constants quantifying the CA exchange rate between plasma and extravascular-extracellular space (EES), respectively. With initial condition $C_{p} (0)=0$, the integration form of Eq. (1) is as follows,

2 $$\begin{aligned} C_{T} (t)=K_{a} \int _{0}^{t} C_{p} (s)e^{-K_{b} (t-s)} ds. \end{aligned}$$

Equation (2) is a commonly used in many applications [24]. Murase [82] suggested another solution of Eq. (1) using discretization:

3 $$\begin{aligned} C_{T} (t)=K_{a} \int _{0}^{t} C_{p} (s)ds-K_{b} \int _{0}^{t} C_{T}(s)ds, \end{aligned}$$

In matrix form, Eq. (3) is

4 $$\begin{aligned} \vec {C}=\vec {A}\times \vec {K}, \end{aligned}$$

in which the matrix $\vec{A}_{ \times 2} = \{A(1), \ldots , A(n)\}'$ with $I=1,2,...,n:$ includes *n* rows which are defined as ${A(I)=(\int _{0}^{t_{I} } C_{p} (s)ds, -\int _{0}^{t_{I} } C_{T} (s)ds),}$ and $\vec{K}=\left( {K_{a} }, {K_{b}}\right) '$ and $\vec{C}_{T}=\left( {C_{T}(t_{1})},{C_{T} (t_{2} )}, {\ldots }, {C_{T} (t_{n})} \right) '.$ The following linear system of equations arising in various tomographic image restoration and reconstruction problems is considered:

5 $$\begin{aligned} Y_{T} (t_{i} )=A(i)\vec {K}+b_{i} \end{aligned}$$

where $b _{i} \mathrm{\sim }N(0,\sigma ^{2})$ and $Y_{tis}(t_{i})$ are, respectively, the measured concentration in tissue at time $t_{i}$ and the measured uncertainty (noise), considered to be additive, white, centered, Gaussian and independent of *K* [83].

The ill-posed inverse problem (IPP) Eq. (5) can be simplified to estimate *K* subject to *A* and *Y*. When the forward solution is determined, the important step is to estimate $\hat{K}$ such that *K* optimizes the related measures, like the least square criterion, $J(k)= {\Vert Y-AK \Vert }^{2}$. However, the model might fit to the data, but due to the ill-posedness of the linear problems, it may not have desired properties [79]. To this end, one can consider some initial prior information regarding errors and the unknowns *K*. The problem can then be handled using general regularization theory and by application of the statistical inference. Two different strategies can be used for this, either information theory and entropy, or Bayesian inference [79].

#### Regularization Methods

Regularization is an appropriate method to find a unique and stable solution to the IIP in Eq. (5), [79]. There are two issues at hand. The first one is that $Y = AK$ has more than one solution and there is a need to know more conditions, for example $\bigtriangleup (K,q)$ to choose that unique solution by

6 $$\begin{aligned} \hat{K}=argmin_{K:AK=Y}\bigtriangleup (K,q), \end{aligned}$$

where *q* is a prior solution and $\bigtriangleup$ a distance measure. The Lagrangian approach [79, 84] has been described as the best method to solve this. With the Lagrangian $L(K,q)=\bigtriangleup (K,q)+ \lambda ^{t}(Y-AK)$ on can estimate $(\hat{\lambda }, \hat{K})$ via

7 $$\begin{aligned} \hat{\lambda }&=argmin_{\lambda }\{ D(\lambda )=inf_{K}L(K,\lambda )\},\\ \hat{K}&=argmin_{K}\{L(K,\hat{\lambda })\}. \end{aligned}$$

or another solution by

8 $$\begin{aligned} \hat{K}=argmin_{K}\{\bigtriangleup (Y,AK)\} \end{aligned}$$

$\bigtriangleup (Y,AK)$ is a measure of distance between *Y* and *AK*. In here, the $\bigtriangleup (Y,AK)={\Vert Y-AK \Vert }^{2}$ is least square (LS) criterion. Obviously, if $\hat{K}$ satisfies $A^{t}A\hat{K}=A^{t}Y$ (the normal equation), it will be a solution to the LS approach. In addition, when $A^{t}A$ is invertible and well-conditioned, then $\hat{K}=(A^{t}A)^{-1} A^{t}Y$ is again the unique generalized inverse solution [79].

#### Bayesian Estimation Approach

An alternative approach is to use Bayesian inference, which allows to find the exact parameter estimations, not only approximations. To this end, the parameters are considered as random variables, with prior distribution using prior information, e.g., from earlier data [85, 86]. Here, we need prior distributions for errors and unknown parameters.

The following equation (Eq. (5)) proposed by [85–88] is considered to estimate the pharmacokinetic parameters. The problem is to estimate the positive-vector *K* (the pixel intensities in an object) under a measured vector *Y* (e.g., a degraded image or the projections of an object) and a linear transformation *A* that links both vectors via

9 $$\begin{aligned} Y=AK+b. \end{aligned}$$

Subject to *p*(*K*), *p*(*Y*|*K*) and *p*(*Y*), the posterior probability distribution of *K* condition to *Y*, *p*(*K*|*Y*), using Bayes: rule will be [89]:

10 $$\begin{aligned} p(K|Y)=\frac{p(Y|K)\cdot p(K)}{p(Y)}. \end{aligned}$$

The Bayesian estimator $\hat{K}$ can be determined by maximizing *p*(*K*|*Y*), such that in Eq. (10), *p*(*Y*) has no dependence on *K*, *p*(*Y*|*K*) is related to noise, and *p*(*K*) is a prior distribution of *K*.

The PDFs *p*(*K*) and *p*(*Y*|*K*) can be estimated using MET as proposed by [85, 86, 89], with the general form of the estimated model belonging to the exponential family. In the MET, initial information to define the constraints of *p*(*K*) is required to choose the model with the maximum entropy (see “Maximum Entropy Technique- Entropy as a Regularization Functional”). The posterior distribution is computed as follows

$$\begin{aligned} p(Y|K) &\approx \exp [-Q(K)],\\ Q(K)&= [Y-AK]^{t} [Y-AK]/\sigma ^{2} . \end{aligned}$$

### Maximum Entropy Technique—Entropy as a Regularization Functional

We propose to solve the ME problem using the regularization method. Generally, there is a unique optimizer to solve either $J(x)={\Vert Y-Ax \Vert }^{2}+\lambda P(x)$ or

11 $$\begin{aligned} J(x)=\bigtriangleup _{1}(Y-Ax)+\lambda \bigtriangleup _{2}(x,q) \end{aligned}$$

where $\bigtriangleup _{1}$ and $\bigtriangleup _{2}$ are two distance measures, $\lambda$ is a regularization parameter and *q* is a priori solution. The important step is to choose $\bigtriangleup _{1}$ and $\bigtriangleup _{2}$, and determining $\lambda$ and *q*. The main part of MET is maximizing Shannon’s entropy [38]:

12 $$\begin{aligned} H(X)=-\int p(x)\ln p(x)dx, \end{aligned}$$

subject to the following constraints, which are the expectations of known functions computed numerically based on the data via Taylor’s theorem [90].

13 $$\begin{aligned} E(\phi _{i} (x))=\int \phi _{i} (x)p(x)dx=\mu _{i}, \end{aligned}$$

in which $\phi _{0} (x)=1$, and $\phi _{i} (x)$, $k=0,\ldots ,N$ are $N+1$ known functions. The general forms of these functions are $x^{n}$,$\ln (x)$, $x\ln (x)$, trigonometric or geometric functions [37]. Entropy can also be used as a regularization functional in Eq. (11). An essential challenge in this method is to specify the regularization parameter $\lambda$. Here,

14 $$\begin{aligned} J(p)={\Vert \mu - E(\phi (x)) \Vert }^2+\lambda D_{K-L}(p,g) \end{aligned}$$

where $D_{K-L}$ is Kullback–Leibler divergence $D_{K-L}$ and *g* is an initial solution of *p*. *J*(*p*) is convex on $R_{+}^{n}$ and if the solution exists, it will be unique. Using the Lagrangian technique gives the following:

15 $$\begin{aligned} \hat{p}_{j}=g_{j}exp[-[A^{t} \hat{\lambda }]_{j}] \end{aligned}$$

with

16 $$\begin{aligned} \hat{\lambda }= argmin_{\lambda }\{ D(\lambda )= \lambda ^{}\mu - G( \phi (A^{t}\lambda ), g ) + \frac{1}{\lambda } {\Vert \lambda \Vert }^2\}. \end{aligned}$$

We mentioned that *p* may be in nonlinear form. In the following, we briefly describe the MET regularization algorithm (MET/REG)

- (1) Assuming $\mu _{i}$s as constraints which are the expected value of the known functions $\phi (x), \in C$, computed numerically from data based on the Taylor’s theorem, $\mu =E_{p}(\phi (X))$.
- (2) Estimating *p*(*x*) by minimizing $D_{K-L}$ subject to the known constraints in Eq. (13). Then, *g*(*x*) is an initial (empirical) solution for *p*. Using the Lagrangian, the following equation is solved
  17 $$\begin{aligned} \begin{aligned} dp(\mu , \lambda )=exp[\lambda ^{t}[Ax]-ln Z(\lambda ) ]dg(x),\\ where~~~~ Z(\lambda )=\int _{C}exp[\lambda ^{t}[Ax]]dg(x) \end{aligned} \end{aligned}$$
  and its parameters will be determined by $\partial ln Z(\lambda )/ \partial \lambda _{i}$, $i= 1, ...,M$.
- (3) Determining the expected value of *p*, $\hat{\mu }(\lambda )=E_{p}(X)=\int xdp(x, \lambda )$ as the solution of the inverse problem. The solution $\hat{\mu }$ is a function of dual variable $\hat{s}= A^{t}\hat{\lambda }$ by $\hat{\mu }(s)= \bigtriangledown _{s}G(\hat{s}, q)$ in which
  18 $$\begin{aligned} \begin{aligned}G(s, q)&= ln Z(s,q)=ln \int _{C}exp [s^{t}x]dg(x) ,\\q&=E_{g}(X)= \int _{C} xdg(x) \\ \hat{\lambda }&= argmax_{\lambda } \{ D(\lambda )= \lambda ^{t}y - G(A^{t}\hat{\lambda })\}. \end{aligned} \end{aligned}$$
- (4) If the function *g* is a separable measure: $g(x)=\prod _{j=1}^{N} g_{j}(x_{j})$ then *p* is a separable measure: $dp(x, \lambda )=\prod _{j=1}^{N} dp_{j}(x_{j}, \lambda )$ and then,
  19 $$\begin{aligned} G(s, q)=\sigma _{j}g_{j}(s_{j}, q_{j}), \end{aligned}$$
  function $g_{j}$ will be the logarithmic Laplace transform of $g_{j}: g_{j}=ln \int exp[sx]dg_{j}(x)$.

### Maximum Entropy Technique—Teaching–Learning-Based Optimization

In the previous work [76, 77], the MET/MAP and MET/TLBO have been applied to estimate the ME distribution of the AIF along with the pharmacokinetic parameters. In the following a brief description of the MET/TLBO is provided.

Applying the Lagrange multipliers approach proposed by [36, 37, 90] where Shannon’s entropy Eq. (12) is a target function with the known constraints as in Eq. (13), *J*(*p*) is

20 $$\begin{aligned} J(p)=-\int p(x)\ln p(x)dx+\lambda _{0} \int p(x)dx+\sum _{i=1}^{N} \lambda _{i} \int p(x)\phi _{i} (x)dx.\, \end{aligned}$$

where *p*(*x*) is determined via differentiating *J* subject to *p*(*x*):

21 $$\begin{aligned} \frac{\partial J(p)}{\partial p(x)} =-\ln p(x)-1+\lambda _{0} +\sum _{i=1}^{N} \lambda _{i} \phi _{i} (x).\, \end{aligned}$$

When setting Eq. (20) equal to zero, *p*(*x*) is as follows [38]:

22 $$\begin{aligned} p(x)=e^{-\sum _{i=0}^{N} \lambda _{i} \phi _{i} (x)},~~ x\in S,{} \end{aligned}$$

and $\lambda _{i}$ are estimated when the determined *p*(*x*) in Eq. (21) satisfies Eq. (13). In addition, determination of $\lambda =[\lambda _{0} ,...,\lambda _{N} ]$ is the cornerstone for the specification of the family of estimated distributions. For that, TLBO is used to solve the $N+1$ unknown parameters, as the set of $N+1$ nonlinear equations a($1\le k\le m$):

23 $$\begin{aligned} G_{i} (\lambda )=\int \phi _{i} (x)e^{-\sum _{i} \lambda _{i} \phi _{i} (x)} dx=\mu _{i} . \end{aligned}$$

The TLBO is a commonly used technique which simulates the teaching–learning process in a class [78]. Here, a group of students are considered to be the target population, and the subjects concerning them are variables of the optimization problem. The scores of students in any subject are the value of the mentioned variables. The teacher is the best solution in the whole population and distributes his information to the students modifying the quality of learning. Additionally, the quality of a student is determined by the average value of the student’s scores in the same class. The algorithm has two main steps:

#### Teacher Phase

Here, the teacher attempts modifying the average scores of the students condition on their situation to produce a new result replacing the old one. This is a random step:

24 $$\begin{aligned} Z_{neu, D}=Z_{Alt, D}+ r(Z_{Te, C}-S_{F}M_{C}) \end{aligned}$$

where *C* is the number of courses, $Z_{Alt, C}$ (a vector $1 \times C$) is the old result with no contribution for the learners to increase their information and involves the results of every specific course, a random number $r\in [0,1]$, $Z_{Te, C}$ gives the most desirable solution in the entire population, $S_{F}$ is a teaching factor ranging randomly from 1 to 2 with the same probability, and vector $M_{C}$ ( $1 \times C$) is the mean scores of the class in any course. The new solution $Z_{neu, C}$ is considered as better than the old one [91].

#### Learner Phase

Here, the aim is to improve the information of each student in situations in which he/she has random cooperation with other students, Eq. (24) is applied to the whole class. This way, a student is able to obtain new information from another student who has more information.

25 $$\begin{aligned} Z_{neu, i}=Z_{ALt, i}+ r_{i}(Z_{j}-Z_{l}) \end{aligned}$$

In the above, $i=1,2$ is the solution number, $Z_{Alt, i}$ means the lack of cooperation between $r_{i} \in [0,1]$, and $Z_{j}$ and $Z_{l}$ represent two students selected randomly for $j\ne l$ when $Z_{j}$ provides a better objective value than $Z_{l}$ . If the solution $Z_{new, i}$ is better than the old one $Z_{Alt, i}$, it is accepted.

### Evaluation Procedure

All computations are done using MATLAB. To determine the general form of the ME distribution, we have applied the Kernel distribution using ‘KernelDistribution’ objects and ‘ksdensity’ in MATLAB. Regularization algorithms considered were Lasso, Ridge regression (Tikhonov regularization), and the generalized minimal residual (GMRES) method. MATLAB functions for these three methods were ‘lasso’, ‘ridge’ and ‘gmres’, respectively. The step-by-step algorithm (regularization of entropy) is provided in “Maximum Entropy Technique- Entropy as a Regularization Functional”. To assess the accuracy of each step of the algorithm, in addition to the Kullback–Leibler divergence $D_{K-L}$, we have used symmetric measurements to evaluate the closeness of the estimated ME distribution to the empirical one. In addition, some other statistics which are robust for the comparison are mentioned:

1. Evaluation by measuring distance between the estimated PDF and the empirical one, e.g., Kullback–Leibler divergence $D_{K-L}$
  26 $$\begin{aligned} D_{K-L} ({\mathop {\hat{p}}\limits ^{}} \mathrm{|}|g)=\int _{s} \hat{p}_{C_{p} } (t) \ln \frac{\hat{p}_{C_{p} } (t)}{g(C_{p} )} dt. \end{aligned}$$
2. Evaluation by using measures comparing the estimated values to the sample data. With the predicted values $\hat{y}_{1} ,...,\hat{y}_{l}$ and the observed values $y_{1} ,...,y_{l}$:
  27 $$\begin{aligned} R-MSE=\Big [\frac{1}{l} \sum _{i=1}^{l}(y_{i} -\hat{y}_{i} )^{2} \Big ]^{1/2} , \end{aligned}$$
  28 $$\begin{aligned} Chi-Square =\frac{\sum _{i=1}^{l}(y_{i} -\hat{y}_{i} )^{2} }{l-n} , \end{aligned}$$
  29 $$\begin{aligned} R^{2} =1-\frac{\sum _{i=1}^{N}(y_{i} -\hat{y}_{i} )^{2} }{\sum _{i=1}^{N}(y_{i} -\bar{y})^{2} } , \end{aligned}$$

## Numerical Experiment

### Data Description

In this study, DCE-MRI images of twelve patients before treatment were used. For each patient, once Gadolinium-DTPA was administered as the CA, 46 scans were taken at intervals of 11.9 seconds. To calculate the values of $T_{1}$, based on calibration curves reported in [92, 93], a two-point measurement was employed. $T_{1}$ in DCE-MRI gives the relaxation time, which measures the recovering rate of the net magnetization vector. The value of $T_{1}$ is calculated as a ratio of a $T_{1}$-weighted fast low-angle shot (FLASH) image and a proton-density-weighted FLASH image. The CA concentration $C_{T} (t)$ is measured by converting the signal intensity into $T_{1}$ using $T_{1}$-weighted and proton-density-weighted images as well as data from calibration phantoms knowing $T_{1}$ [94]. The concentration of Gd-DTPA is calculated by $C_{T} (t)=\frac{1}{r_{1} } \left[ \frac{1}{T_{1} (t)} -\frac{1}{T_{10} } \right] ,$ where $T_{10}$ is the $T_{1}$ with no contrast, calculated as the average of the first four images, and $r_{1} =4.24{} \mathrm{l/s/mmol}$ gives the in vivo longitudinal relativity of protons from Gd-DTPA. For the T1-weighted FLASH images, the obtained parameters are TR = 11 ms, TE = 4.7 ms, $\alpha$ = 35, with the corresponding parameters for the proton-density-weighted images are TR = 350 ms, TE = 4.7 ms, $\alpha$ = 6. All the scans had the same field of view, namely $260\times 260\times 8$ mm per slice, making the voxel dimensions of $1.016\times 1.0168$ mm. Each scan included three successive slices with $256\times 256$ voxels and one slice put in the contra-lateral breast as control, which was not used for this analysis. Following the fourth scan, Gd-DTPA was injected at D = 0.1 mmol per kg body-weight using a power injector with 4 mL/s with a 20 mL saline flush also at 4 mL/s.

### Example Description

**Fig. 1:**
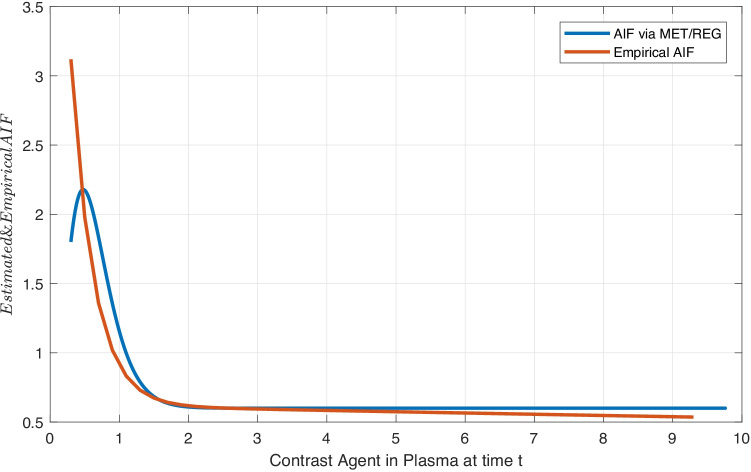
Estimated AIF via MET/REG. (Gamma Distribution) & Empirical AIF

We compare the proposed MET/REG algorithm with the previously proposed MET/MAP and MET/TLBO algorithms. In previous work, the Weibull distribution as PDF for the AIF turned out to satisfy most of the conditions. See Table 1 for more information about the model and estimation approaches. The PDF of the Weibull distribution with two parameters is as follows:

30 $$\begin{aligned} p(x)=\frac{\alpha }{\beta ^{\alpha } } x^{\alpha -1} exp({-(\frac{x}{\beta } )^{\alpha } }) , \end{aligned}$$

where $\alpha$ and $\beta$ are the shape and scale parameters, respectively [95, 96]. The MET tests and utilizes different moment constraints [37] and selects the minimum number of them to generate a proper PDF of the observation. The moment constraints as known functions are based on the data, and their expectations are obtained numerically via the Taylor’s theorem using the observations [90]. Note that adding more constraints does not guarantee a better ME model. The estimated PDF of the data with the initial conditions ($\phi _{0}=1$, $\phi _{1}=\ln (C_{p}(t))$ and $\phi _{2}=C_{p}(t)$) and expected values ($1, -0.446$ and 0.335) from Taylor’s theorem fits well with the Weibull and with the family of Gamma distributions based on MET/TLBO and MET/REG (Fig. 1), respectively. For data $\vec{C}_{p} (t)$ both models are as follows:

31 $$\begin{aligned} p^{(1)}_{C_{p}}(t)&= e^{-\lambda _{0} -\lambda _{1}\phi _{1}(C_{p} (t))-\lambda _{2}\phi _{2}(C_{p} (t)) }, \\&= e^{\ln (\frac{\alpha }{\beta ^{\alpha } } )+(\alpha -1)\ln (C_{p} (t))-\left(\displaystyle \frac{C_{p} (t)}{\beta } \right)^{\alpha }}. \end{aligned}$$

in which $\lambda _{0}=-\ln (\alpha \setminus {\beta ^{\alpha }})$, $\lambda _{1}=-(\alpha -1)$ and $\lambda _{2}=\beta ^{-\alpha }$. Resulting values for $\alpha$ and $\beta$ can be found in Table 2.

**Table 1:** Parameter Estimation Techniques

***Methods***	***Parameter Estimation***
**Empirical Measurement Approach** is the specific case of the method of moment [97] with the Gamma function $\Gamma (.)$.	$$\left\{ \begin{array}{l} {\alpha =(\frac{\sigma }{\bar{x}} )^{-1.086} ,} \\ {\beta =\frac{\bar{x}}{\Gamma (1+\frac{1}{\alpha } )} ,\, \, \, \, \, \, \, \, \, \Gamma (x)=\int _{0}^{\infty }t^{x-1} e^{-t} dt } \end{array}\right.$$
**The Maximum Likelihood Approach** is applied to describe the time-series data with the Weibull distribution [96]. *n* is the size of non-zero data vector, with ($\alpha , \beta$) the shape and scale parameters.	$$\left\{ \begin{array}{l} {\alpha =\Big (\frac{\sum _{i=1}^{n}x_{i}^{\alpha } \ln (x_{i}) }{\sum _{i=1}^{n}x_{i}^{k}} -\frac{\sum _{i=1}^{n}\ln (x_{i})}{n}\Big )^{-1},} \\ {\beta =(\frac{1}{n} \sum _{i=1}^{n}x_{i} {}^{\alpha } )^{1/\alpha } ,} \end{array}\right.$$
**Modified Maximum Likelihood Approach** is utilized when data has the frequency distribution form. $P(x_{i})$ is the data $x_i$, *n* the number nonzero data, and $P(x\ge 0)$ the probability of the random variable equal or exceeding zero and $\alpha$ and $\beta$ are determined explicitly [98, 99].	$${\left\{ \begin{array}{ll}\alpha =\left( \frac{\sum _{i=1}^{n}x_{i}^{\alpha } \ln (x_{i}) P(x_{i})}{\sum _{i=1}^{n}x_{i}^{\alpha } P(x_{i})} -\frac{\sum _{i=1}^{n}\ln (x_{i} )P(x_{i} )}{{P(x\ge 0)}} \right) ^{-1},\\ \beta =\left( \frac{1}{P(x\ge 0)} \sum _{i=1}^{n}x_{i}^{\alpha } P(x_{i})\right) ^{1/\alpha }, \end{array}\right. }$$

**Table 2:** Estimated parameters for different approaches

***Estimation Approach***	$\varvec{\alpha }$	$\varvec{\beta }$
Empirical (Weibull)	1.647	0.779
Maximum Likelihood (Weibull)	1.801	0.789
Modified Maximum Likelihood (Weibull)	2.020	0.776
Maximum Entropy & MAP (Exponential)	–	1.554
Maximum Entropy & TLBO (Weibull)	2.6	1.738
Maximum Entropy & REG. (Gamma)	2.719	0.237
Maximum Entropy & REG.(Erlang)	2	0.237

The general form of the Gamma distribution and its ME model are:

32 $$\begin{aligned} p^{(2)}_{C_{p}}(t)&= e^{-\lambda _{0} -\lambda _{1} \ln (C_{p} (t))-\lambda _{2}C^{}_{p} (t) }, \\&= e^{\ln (\frac{1}{\Gamma (\alpha )\beta ^{\alpha } } )+(\alpha -1)\ln (C_{p} (t))-\left(\displaystyle \frac{C_{p} (t)}{\beta } \right)}. \end{aligned}$$

in which $\lambda _{0}=-\ln (1 \setminus \Gamma (\alpha ){\beta ^{\alpha }})$, $\lambda _{1}=-(\alpha -1)$ and $\lambda _{2}=\beta ^{-1}$. For Erlang distribution,

33 $$\begin{aligned} p^{(3)}_{C_{p}}(t)&= e^{\ln (\frac{1}{(\alpha -1)!\beta ^{\alpha } } )+(\alpha -1)\ln (C_{p} (t))-\left(\displaystyle \frac{C_{p} (t)}{\beta } \right)}. \end{aligned}$$

in which $\lambda _{0}=-\ln (1 \setminus (\alpha -1)!{\beta ^{\alpha }})$, $\lambda _{1}=-(\alpha -1)$ and $\lambda _{2}=\beta ^{-1}$. Based on Eqs. (30) to (33), the estimated parameters for all models are presented in Table 2, respectively.

**Fig. 2:**
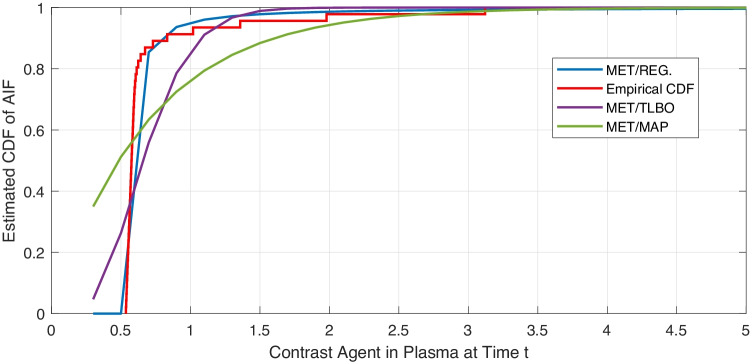
Estimated CDFs of AIF via MET/MAP, MET/TLBO and MET/REG.-Gamma Distribution & eCDF of AIF

**Fig. 3:**
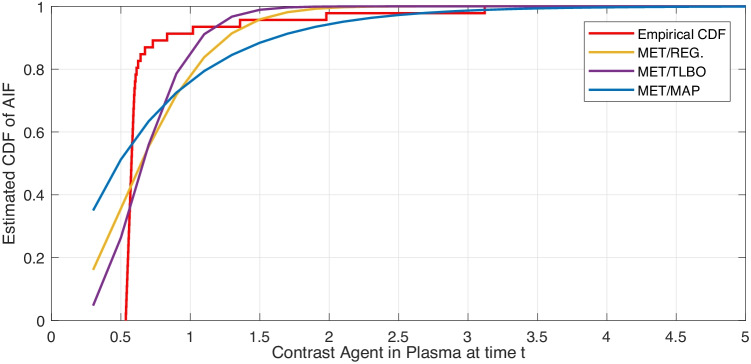
Estimated CDFs of AIF via MET/MAP, MET/TLBO and MET/REG.-Erlang Distribution & eCDF of AIF

Table 3 shows Kullback–Leibler distance $D_{K-L}$ and the entropy of different estimated AIFs for model evaluation. Higher values of entropy indicate better AIF estimation. The value of $D_{K-L}$ is the distance between the estimated AIF and the empirical one. Lower values means the two models are close together. Figures 2 and 3 show the curve of CDFs from all proposed approaches and the empirical CDF for visual validation. In Table 4, the results are evaluated via *RMSE*, the goodness of fit ($\chi ^2$), and determination coefficient ($R^{2}$).

**Table 3:** Evaluation scales to compare the estimated and the empirical AIFs

**Method & Model**	${\boldsymbol {K}-\boldsymbol {L}}$ Divergence	**Entropy**
MET/MAP-EXP.	0.029	0.031
MET/TLBO/MAP-WB. $(\alpha =2)$	0.036	0.087
MET/TLBO/MAP-WB. $(\alpha =3)$	0.044	0.21
MET/TLBO/MAP-WB. $(\alpha =2.6)$	0.039	0.176
MET/REG.-GAMMA.	0.040	0.097
MET/REG.-ERLANG.	0.029	0.103

Tables 3 to 4 show that MET/TLBO and MET/REG perform the best. The proposed novel MET/REG has the lowest $D_{K-L}$ divergence and high entropy.

**Fig. 4:**
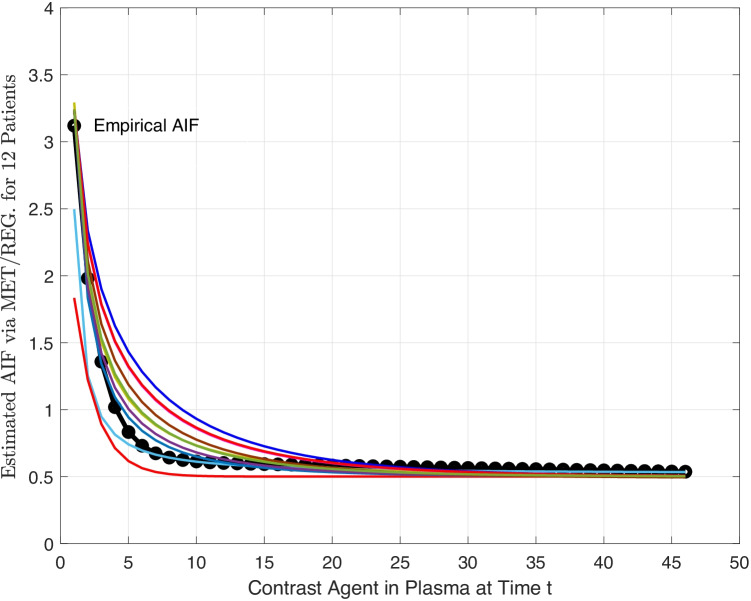
Estimated PDFs of AIF via MET/REG. for 12 Patients

**Fig. 5:**
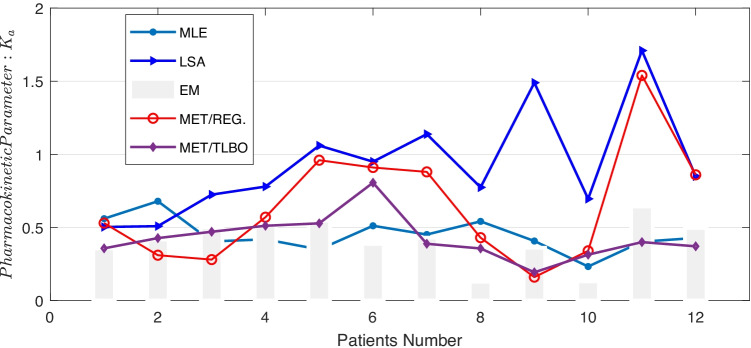
Estimated Pharmacokinetic Parameters via different Approaches

For all 12 patients, similar results in regard to model performance are achieved, see Fig. 4). Nevertheless, to obtain a proper estimation of the pharmacokinetic parameters, correct estimation of the AIF is of particular importance. The measured $D_{K-L}$ values are in the range (0, 0.1) for all 12 patients. In Fig. 5, $K_{a}$ estimations are provided based on MET/REG and assumed AIF/ML & MET/MAP and MET/TLBO for all the patients. Most importantly, using the estimated AIF via MET/REG led to more realistic *k* values compared to assumed AIF, see Fig. 5 and Table 5.

**Table 4:** Evaluating Approaches for the MET/REG model of AIF and the empirical one

***Methods***	***Root-MSE***	***Chi-Square***	$\boldsymbol {R}^\mathbf {2}$
Empirical Approach	0.29	0.08	0.63
Maximum Likelihood Approach	0.28	0.12	0.57
Modified Maximum Likelihood Approach	0.28	0.08	0.64
Maximum Entropy Approach & TLBO	0.03	7.5e-04	0.99
Maximum Entropy Approach & Regularization-Gamma	0.07	0.10	0.99
Maximum Entropy Approach & Regularization-Erlang	0.07	0.1	0.97

**Table 5:** Estimated Pharmacokinetic parameters via MET/REG

**Patient**	**1**	**2**	**3**	**4**	** 5**	**6**	**7**	**8**	**9**	**10**	**11**	**12**
$k_{a}$	0.53	0.31	0.28	0.57	0.96	0.91	0.88	0.43	0.16	0.34	1.54	0.86

##  Discussion and Conclusions

In this paper we investigated the application of MET and regularization functionals with some probabilistic models to solve the problem of AIF determination in DCE-MRI, a linear ill-posed inverse problem. Reasons for applying the MET in combination with the selected optimization or regularization methods to estimate the AIF, instead of using the assumed AIF, were discussed. In addition, the effect of the estimated AIF on the determination of pharmacokinetic parameters was examined.

We have shown how all these different frameworks converge to solve the linear ill-posed inverse problems. The results show that the Bayesian framework provides more tools to infer the uncertainties of the computed solutions, account for more specific knowledge of the noise and errors, estimate the hyper-parameters, and handle myopic and blind inversion problems. For that, regularization, MET, and the Bayesian estimation approach were discussed briefly. Finally, we presented some numerical results to illustrate the efficiency of the presented method. The main objective of these numerical experiments was to demonstrate the effect of different choices for prior laws or, equivalently, regularization functionals on the result. To determine the ME solution via entropy regularization, it was assumed that the existing data are represented by generalized moments, including the power and the fractional ones as a subset. However, as mentioned in the paper, the solution of an inverse problem generally depends on our prior hypothesis regarding AIF, errors and *K*.

The proposed MET/REG algorithm has multiple notable features: (1) applicability to distributions with any type of support, (2) efficiency in terms of computation since the ME solution is derived simply as a set of linear equations, (3) proper bias-variance, and (4) proper estimation of the distribution tails when the sample sizes are small. Given the important role of the AIF when analyzing DCE-MRI images, when determining the AIF in the image is not possible, a standard approach is to use assumed AIFs proposed in the literature. This research provides an alternative method for the assessment of the AIF from the available information. Based on the results, the estimated model using MET/REG fits well to the data and properly estimates the pharmacokinetic parameters.

## Data Availability

There would be possible by personal request. Breast cancer data set provided by Paul Strickland Scanner Centre at Mount Vernon Hospital, Northwood, UK Scanner Centre at Mount Vernon Hospital, Northwood, UK

## Code Availability

There would be possible by personal request.
